# Divergent responses of viral and bacterial communities in the gut microbiome to dietary disturbances in mice

**DOI:** 10.1038/ismej.2015.183

**Published:** 2015-10-16

**Authors:** Adina Howe, Daina L Ringus, Ryan J Williams, Zi-Ning Choo, Stephanie M Greenwald, Sarah M Owens, Maureen L Coleman, Folker Meyer, Eugene B Chang

**Affiliations:** 1 Iowa State University, Ames, IA, USA; 2 Argonne National Laboratory, Argonne, IL, USA; 3 Department of Medicine, University of Chicago, Chicago, IL, USA

## Abstract

To improve our understanding of the stability of mammalian intestinal communities, we characterized the responses of both bacterial and viral communities in murine fecal samples to dietary changes between high- and low-fat (LF) diets. Targeted DNA extraction methods for bacteria, virus-like particles and induced prophages were used to generate bacterial and viral metagenomes as well as 16S ribosomal RNA amplicons. Gut microbiome communities from two cohorts of C57BL/6 mice were characterized in a 6-week diet perturbation study in response to high fiber, LF and high-refined sugar, milkfat (MF) diets. The resulting metagenomes from induced bacterial prophages and extracellular viruses showed significant overlap, supporting a largely temperate viral lifestyle within these gut microbiomes. The resistance of baseline communities to dietary disturbances was evaluated, and we observed contrasting responses of baseline LF and MF bacterial and viral communities. In contrast to baseline LF viral communities and bacterial communities in both diet treatments, baseline MF viral communities were sensitive to dietary disturbances as reflected in their non-recovery during the washout period. The contrasting responses of bacterial and viral communities suggest that these communities can respond to perturbations independently of each other and highlight the potentially unique role of viruses in gut health.

## Introduction

The impact of the mammalian gut microbiome on health and disease, especially its response to diet, has gained increasing attention in recent years. Previous studies have demonstrated that the community membership and diversity of bacterial communities shift rapidly with diet-specific responses in mice (Turnbaugh, Ridaura, *et al.*, 2009; Zhang *et al.*, 2012) and in humans (Turnbaugh, Ridaura, *et al.*, 2009; Walker *et al.*, 2010; Zhang *et al.*, 2012; David *et al.*, 2013). These gut bacterial communities have been shown to be resistant to dietary changes, recovering their pre-intervention structure once the host resumes a previously consumed diet (Zhang *et al.*, 2012; David *et al.*, 2013). Alongside bacteria, viruses, mainly bacteriophages, are also abundant in the gut (Breitbart *et al.*, 2008; Reyes *et al.*, 2010; Minot *et al.*, 2011; Norman *et al.*, 2015) and have been hypothesized to markedly alter bacterial community structure and function through gene exchange, eliminating niche competitors and modification of gene expression of hosts (Reyes *et al.*, 2012; Duerkop *et al.*, 2012; Barr *et al.*, 2013). Despite their abundance and potential to impact gut community dynamics, little is known about the overall impact of viruses on the stability and function of the gut microbiome. Recently, enteric viruses of stool filtrates were found to be disease- and cohort-specific in patients with Crohn's disease and ulcerative colitis (Norman *et al.*, 2015). Other studies on viruses in human gut microbiomes have suggested that they are largely stable within an individual yet variable within the population (Reyes *et al.*, 2010; 2012; Minot *et al.*, 2012). Short-term diet interventions have been shown to alter viral communities in humans (Minot *et al.*, 2011), but the lasting impact of dietary shifts on viruses and gut health and their resistance to disturbances is unknown.

Given their ability to markedly alter other ecosystems, there is reason to hypothesize that viruses are drivers of ecosystem function in the gut. In the ocean, cycles of lytic phage infection have been demonstrated to profoundly impact the structure of microbial communities (for example, population shifts through cell lysis) and the availability of metabolic functions (for example, gene transfer through infection) (Suttle, 2007; Rohwer and Thurber, 2009; Gomez and Buckling, 2011). Unlike marine phages, which are believed to be mostly lytic, recent efforts indicate that the gut appears to host a largely temperate phage-bacteria dynamic (Reyes *et al.*, 2010; Minot *et al.*, 2011). The impact of this largely uncharacterized and potentially persistent community is unknown yet critical toward understanding the dynamics of the gut microbiome and managing its impact on health.

To better understand the dynamics of gut viruses alongside bacterial communities, we characterized the concurrent responses of bacterial and viral intestinal communities in mice that underwent a dietary perturbation. Mice were fed a purified low-fat (LF) or high-milkfat (MF) diet, switched to the alternate diet, and returned to the original diet for a washout period to study the resistance of community responses. The selection of the high-refined sugar, MF diet was based on its approximation of Western consumption (CDC, 2004). It is a high-saturated fat diet (37.5% total kCal) and has previously been shown to perturb the mouse intestinal microbiome (Devkota *et al.*, 2012). As obesity-inducing diet-associated modifications in the gut microbiome have been shown to mediate conditions such as obesity and diabetes (Ley, 2005; Turnbaugh *et al.*, 2006; 2008), we were interested in observing how the gut microbiome, especially the virome, may respond to a high-sugar, high-saturated fat diet. We used metagenomes representing bacterial communities and extracellular viruses and prophages to assess the responses and resistance of bacteria and viruses to varying dietary regimes and disturbances.

## Materials and methods

### Animals

C57BL/6 mice were bred and maintained under standard 12:12 h light/dark conditions at the University of Chicago. Co-housed female mice from mixed litters (*n*=3 per group) were fed purified high fiber, LF (Teklad TD.00102, Harlan Laboratories, Indianapolis, IN, USA) or purified high-refined sugar, MF diet (Teklad TD.97222, Harlan Laboratories) (Table 1) *ad libitum* after weaning (~3-4 weeks of age). At 8–9 weeks of age (Experimental Day 0) the diets of the mice were disturbed with a switched diet for 3 weeks, followed by a return to their original diets for three weeks. Stool samples were collected weekly. All animal protocols and experiments were approved by the Institutional Animal Care and Use Committee at the University of Chicago.

### DNA isolation for BAC, VLP and IND metagenomes

Stool was collected weekly from mice, from which DNA was directly extracted for 16S ribosomal RNA (rRNA) gene amplification and for bacterial (BAC) metagenomic libraries. Viruses in the stool were targeted in two separate fractions: virus-like particles (VLPs) and inducible prophages (IND) present in gut bacteria. DNA originating from virus-like particles (VLP) was isolated from 0.22 μm-filtered supernatants of stool slurries followed by purification on an iodixanol (Optiprep, Sigma Aldrich, St Louis, MO, USA) density gradient. DNA representing IND was obtained from VLPs collected from stool pellet slurries (from which VLPs were already removed) incubated anaerobically with mitomycin C for 18 h at 37 °C. Owing to low sample yield, it was necessary to amplify DNA from viral fractions (VLP and IND) with whole-genome amplification (GenomiPhiV2; GE Lifesciences, Pittsburgh, PA, USA). DNA isolation, extraction, and quantification methods are described in more detail in Supplementary Methods.

### DNA sequencing

Amplified products of the V4–V5 region of the 16S rRNA gene originating from the BAC fraction were sequenced with Illumina MiSeq (average read length 151 bp, Argonne National Laboratory, Argonne, IL, USA) using barcoded primers 515F/806R (Caporaso *et al.*, 2012). The resulting single-end sequences were analyzed with the Quantitative Insights into Microbial Ecology (QIIME) toolkit (Caporaso *et al.*, 2010) (MG-RAST IDs: 4569071.3-4569118.3). Paired-end metagenome sequencing libraries were prepared for BAC, VLP and IND DNA with the Illumina HiSeq2000 (average read length 101 bp, Argonne National Laboratory). Metagenomes were assembled as described in (Pell *et al.*, 2012) and (Howe *et al.*, 2014). These metagenomes and their assembly data sets are publicly available in MG-RAST (MG-RAST-IDs: 4535377.3, 4535378.3, 4535380.3–4535403.3, 4535405.3–4535427.3, 4535523.3, 4535747.3–4535755.3, 4535915.3, 4535927.3–4535934.3). Assembled sequences >200 bp were annotated using the Metagenomics RAST (MG-RAST, v3.3.7.3, (Meyer *et al.*, 2008)) server, NCBI RefSeq (Release 66) and MetaVir2 (Roux *et al.*, 2014). Contig abundances were estimated as the relative coverage, or the median bp coverage of each contig divided by the total median bp coverage of all contigs in each sample. Abundances were compared between diet treatments, days of the experiment and originating DNA extraction fractions. Analyses are described in more detail in Supplementary Methods.

## Results

### No observable difference in the physical response of mice to diet shifts

**Figure 1:**
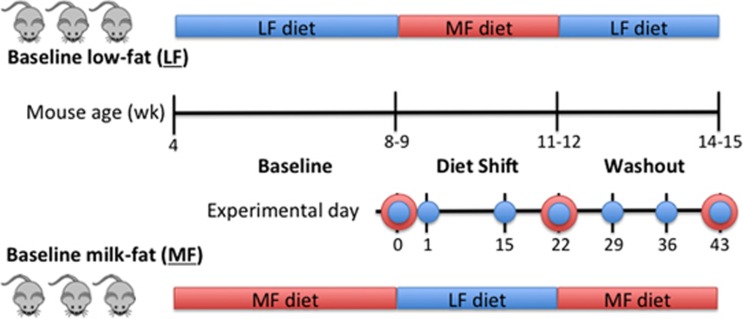
Study design of two mouse cohorts (*n*=3) with varying diet treatments (baseline low-fat (LF) and high-milkfat (MF) diets). Weekly fecal samples were collected for microbial community composition (16S rRNA amplicon analysis, blue) and function (metagenomic sequencing, red).

The two groups of mice were fed LF or MF diet for 4–5 weeks prior to the start of the experiment (Day 0), followed by 3 weeks of the other diet (the dietary perturbation, through Day 22), and 3 weeks of their initial baseline diet (washout, through Day 43; Figure 1). Despite differences in baseline diets, no significant differences in the weight gain of the mice (Supplementary Figure S1) over the course of the experiment were observed.

### Characterization of BAC, IND and VLP metagenomes

For each DNA fraction (BAC, IND, VLP), all associated metagenomes were cumulatively assembled, providing deeper sequencing depth for assembly. The resulting assembled contigs from each fraction were combined, and contigs sharing >99% sequence similarity were clustered, with the longest contig selected as representative. The sequencing efforts for this study purposefully targeted deep sequencing of DNA from viral fractions (Supplementary Table S1), and we observed the most reads incorporated into assembled contigs from viral fractions (Supplementary Table S2). We generated a total of 210 580 Mbp from 65 samples in this study (Table 2), and these reads assembled into a final representative assembly of 100 Mbp and 117 460 contigs (Table 3 and Supplementary Table S3). The abundances of contigs (from reads mapped) generated in this experiment were estimated for each sample. To allow comparisons between samples with different sequencing depths, contig coverage in each sample was divided by the total coverage of all contigs present in that sample. The resulting relative abundances of assembled sequences present in each fraction were compared over the course of the diet study.

Assembled contigs were annotated for both taxonomy and function with homology to their closest representative using MG-RAST and its M5NR SEED database (version 2011-02-22, BLAST-like alignment tool (BLAT), percent identity minimum ⩾60%, minimum *E*-value⩽1e–5), viruses contained with NCBI RefSeq (Release 66) (BLAST, alignment length⩾30 aa, E-value⩽1e–5) and MetaVir2 (which also uses the NCBI RefSeq virus database (Roux *et al.*, 2014), BLAST, *E*-value⩽1e–3). The majority of contigs, though abundant in our samples, could not be annotated against available databases. Among the total 117 460 contigs, 23 811 (21%) could be associated with functional gene annotations and 21 119 (19%) could be associated with taxonomic classifications in MG-RAST (per-sample annotations shown in Supplementary Table S1). Similar proportions of identified sequences with either functional or taxonomic annotations were found in other databases: 20.3% in the NCBI RefSeq viruses and 19.9% in MetaVir. Unless otherwise specified, MG-RAST annotations were used in the remainder of this study.

**Figure 2:**
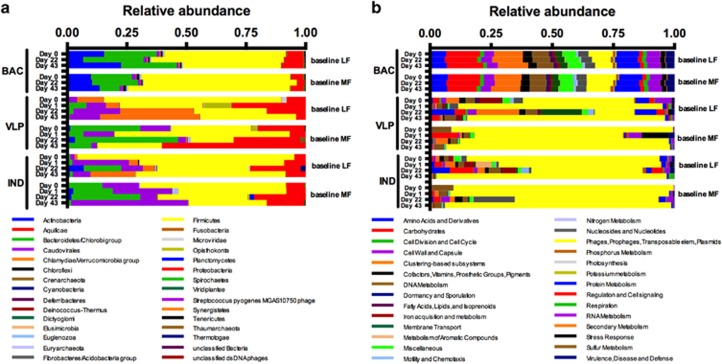
Standardized relative abundances (abundances standardized by total number of reads for each sample) of annotated contigs, which could be assigned to (**a**) taxa or (**b**) functions by MG-RAST M5NR SEED database.

Metagenomes from samples originating from BAC fractions were dominated by bacteria-associated annotations, with the most abundant phyla being Firmicutes, Bacteroidetes, Actinobacteria, Proteobacteria, though hits to the bacteriophage taxon Caudovirales were also present (Figure 2a,Supplementary Table S4). Communities from both baseline LF and MF mice shared broadly similar functional profiles, with the most abundant genes related to carbohydrate, protein and phage metabolism (Figure 2b, Supplementary Table S5). The viral fractions (VLP and IND) of both groups of mice (baseline LF and MF mice) were dominated by sequences related to phage metabolism, many of which were Firmicutes-associated prophage genes.

### Phylogenetic and functional responses of baseline LF- and MF-associated bacterial communities

**Figure 3:**
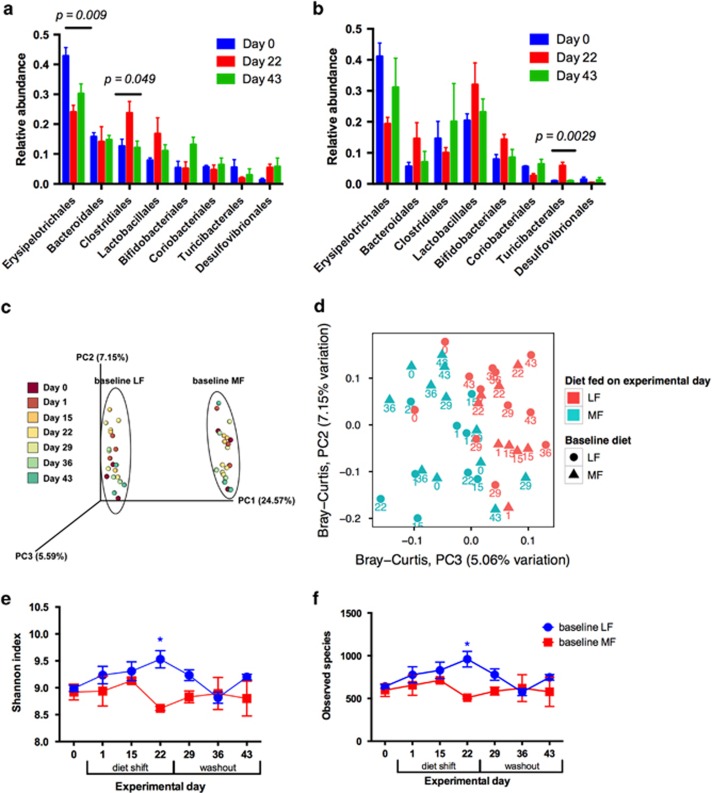
Analysis of bacterial community structure through 16S rRNA genes. Mean relative abundances of order level taxa in fecal bacterial communities present at >1% in (**a**) baseline LF and (**b**) baseline MF mice. Data are shown as mean±s.e.m.; *P*-values (ANOVA) as shown on graph. (**c**) Principal coordinate analysis plot of the bacterial community structure based on Bray–Curtis distances. Symbols represent individual mice, coded by color to represent collection day. (**d**) Principal coordinate analysis of second and third principal coordinate axes, based on Bray–Curtis distances. Colored symbols are the diet the mice were consuming on each experimental day, denoted as the number below each symbol. Changes in alpha diversity of fecal bacterial communities during dietary perturbation and washout: (**e**) Shannon diversity index values and (**f**) number of observed species. Data are shown as mean±s.e.m., *P*-values are shown *P<0.05, **P<0.01, ***P<0.001, ****P<0.0001 comparing the means of both treatment groups at each time point; ANOVA, Bonferroni's multiple comparison test.

To assess the response of bacterial communities to dietary perturbations, we used two data sets: amplicon libraries generated from the V4–V5 region of the 16S rRNA gene in DNA from fecal samples obtained weekly from each mouse during the experiment and BAC metagenomes obtained on Day 0, 22 and 43. In total, 760 470 amplicon reads (15 843±5,486 per sample) were obtained. For both baseline diets, fecal bacterial communities were predominantly composed of members of the phyla Firmicutes (16S rRNA: baseline LF, 75.7±4.41% baseline MF, 84.6±3.35% (mean rel. abundance and s.e.m.)) and Bacteroidetes (16S rRNA: baseline LF, 15.8±2.28% baseline MF±2.29% (mean rel. abundance and s.e.m.), Supplementary Figures S2–S3; BAC metagenomes: Figure 2a and Supplementary Table S4). Baseline communities of LF- and MF-fed mice (Experimental Day 0) clustered separately in a principal coordinates analysis (PC1 and PC2, Figure 3c), reflecting a combination of separate cages and distinct diets after weaning. After dietary perturbation, the compositions of fecal bacterial communities of both groups of mice were altered and appeared to shift by diet along principal component axis 3 (Supplementary Figure S4) and also observed to cluster by diet fed on experimental day (PC2 and PC3, Figure 3d). Taxa shifts within baseline LF mice included a significant decrease in the relative abundance of Erysipelotrichales (analysis of covariance; ANOVA, *P*=0.009) and a significant increase in Clostridiales (ANOVA, *P*=0.049) in 16S rRNA gene amplicons 3 weeks after the switch to the MF diet (Day 22) (Figure 3a). In contrast, in baseline MF mice, a significant increase in Turicibacterales (ANOVA, *P*=0.0029) in 16S rRNA gene amplicons (Figure 3b) was observed (Day 22) after 3 weeks switched on the LF diet. These trends were not observed in BAC metagenomes, presumably given the higher resolution of phylogenetic characterization provided by the greater sequencing depth of our 16S rRNA gene amplification. Although community structure changed (Figure 2a), we did not observe significant differences between functional subsystems on experimental days 0, 22 and 43 in BAC metagenomes in either baseline LF or MF samples (Figure 2b).

Alpha diversity of the fecal bacterial communities fluctuated throughout the experiment with distinct responses for each diet treatment. Bacterial communities from baseline LF mice showed a maximum increase in alpha diversity by Day 22, the end of the switch to MF diet, and were significantly more diverse than the baseline MF group on the same day (Shannon diversity index, two-way ANOVA, Bonferroni, *P*=0.0058, Figure 3e). Bacterial richness (number of observed species) mirrored Shannon diversity in the two diet treatments, and the number of observed species was significantly higher in the baseline LF group on Day 22 than in the baseline MF group (Two-way ANOVA, Bonferroni, *P*=0.0085, Figure 3f).

### Contrasting resistance of bacterial and viral communities to dietary disturbances

**Figure 4:**
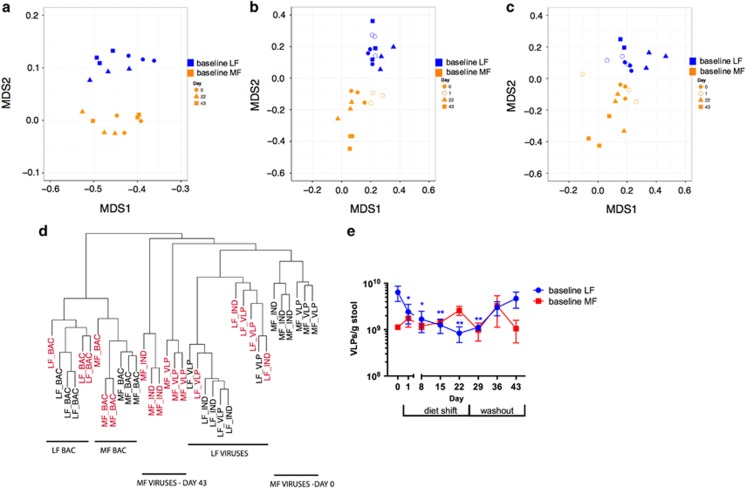
NMDS of Bray–Curtis distances of sequence abundances in (**a**) BAC, (**b**) VLP and (**c**) IND metagenomes. Marker colors represent different diet treatments (orange=baseline MF, blue=baseline LF) and marker shapes indicate day of sample. (**d**) Average similarity hierarchical clustering of BAC, IND and VLP metagenomes on Day 0 (black, baseline) and Day 43 (red, washout) for baseline LF and MF samples, based on Bray–Curtis distances of median bp coverage of sequences. (**e**) Direct counts of VLPs from VLP fraction. Mean VLP counts±s.e.m. are shown. *P<0.05, **P<0.01.

The resistance or sensitivity of bacterial communities to diet disturbances was assessed by comparing the 16S rRNA gene amplicons and BAC metagenome communities on Days 1 (only 16S rRNA amplicons), 22 and 43 to Day 0 communities (Bray–Curtis distance matrix; ADONIS). With only one exception (Day 22, baseline LF, BAC metagenomes), bacterial communities on days 1, 22 and 43 were not significantly different relative to Day 0, as measured by clustering of communities by either 16S rRNA amplicons or BAC metagenomes (Figure 4a, Table 4). By contrast, all of the baseline MF viral communities, both VLP and IND, were significantly different on Day 22 and Day 43 compared with Day 0 (ADONIS, Table 4). This result is consistent with NMDS of baseline MF viral communities, in which samples cluster by day of experiment (Figures 4b and c, Supplementary Figure S5). Comparing Day 0 and 43 communities, bacterial communities from both diets were more similar to each other than viral communities. Further, viral communities from baseline MF mice followed a markedly different trajectory than either the LF viral community or BAC communities from either diet treatment.

Direct counts of VLPs (for example, total free viral particles) isolated from stool confirmed the distinct responses of viruses under baseline MF and LF conditions. VLP counts differed significantly among treatment groups at the start of the experiment (ANOVA, *P*=0.009) (Figure 4e), with baseline LF VLP counts higher than those from baseline MF mice. Unlike viral communities, which changed more strongly in baseline MF mice than in baseline LF mice, VLP abundance responded sharply to diet shifts in baseline LF mice but not in baseline MF mice. In baseline LF mice, VLP abundance decreased significantly during the dietary perturbation relative to day 0 (days 8, 15, 22; ANOVA *P*<0.05) and returned to initial levels by the end of the washout period (Days 36 and 43, ANOVA, *P*>0.05). VLP counts of baseline MF stool showed no clear pattern in response to diet.

To identify functions associated with the response of baseline MF viral communities, contigs whose abundances changed significantly over the course of the experiment were identified (Supplementary Table S7). The most significantly different contigs on days 0, 22 and 43 were identified in the subsystem associated with ‘Phages, prophages, transposable elements and plasmids.' A total of 33 phage subsystem-associated contigs (Supplementary Table S7) exhibited significant abundance changes over the course of the experiment in baseline MF virus fractions, and these contigs could be grouped based on their abundance profiles into two distinct clusters, arbitrarily named Group I (12 contigs) and II (21 contigs) (Bray–Curtis hierarchical clustering, Supplementary Figure S6). PCR amplification of sequences contained in these assembled contigs validated their assembly and their presence in DNA samples (see Supplementary Methods).

The predicted taxonomic origin associated with these contigs varied and included both bacterial hosts and bacteriophages (Supplementary Table S7). The majority of Group I contigs (*n*=7) were associated with Firmicutes, mainly with the taxa *Bacillus* and *Clostridium*, and contained genes associated with phage integration and excision. In Group II, Caudovirales-associated contigs were identified, mainly those similar to Siphoviridae and Myoviridae and with annotations to phage with hosts *Lactobacillus*, *Streptococcus*, *Staphylococcus*, *Clostridium* and *Listeria*.

The relative abundances of Group II contigs are observed to share consistent trends in the baseline MF IND samples, increasing in abundance on Day 1 relative to baseline Day 0 (1.8–3.9%, mean), decreasing in abundances after the diet disturbance (Day 22, 0.09%, mean) and increasing when returned to the baseline diet (Day 43, 8.8%, mean) (Supplementary Figure S7). These trends were not observed in the baseline MF VLP samples, where the majority of these contigs present at baseline were not detectable during Day 1, detected during day 22 and decreased in relative abundance by day 43 (Supplementary Figure S8). On average, Group II contigs were not abundantly present in baseline LF mice (<0.01%) (Supplementary Figure S9), with the exception of the IND fractions, whereas on the MF diet (day 1, 0.9% and day 22, 0.1%, average) (Supplementary Figure S9). In contrast to contigs associated with Group II, trends of Group I contigs are not as clear (Supplementary Figure S6). These contigs decreased throughout the study in IND fractions (0.9–0.1%), and many Group I contigs are only detected on Day 1 and 43 in baseline MF IND samples.

**Figure 5:**
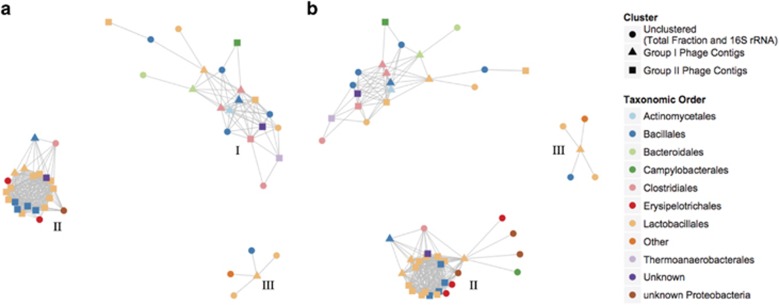
Co-occurrence network representing relationships between significant contigs identified in baseline MF virus fractions (see Supplementary Table S7) and 16S rRNA amplicons and BAC metagenome contigs. Taxonomic orders of sequences that share the most similarity in the MG-RAST database are shown for contigs in the baseline MF VLP (**a**) and IND (**b**) samples. Three separated co-occurrence networks have been labeled and identified as network modules.

To explore potential interactions with bacterial hosts, we tested co-occurrence relationships between these Group I and II contigs and bacterial taxa using BAC-associated contig abundances and 16S rRNA amplicons (see Supplementary Methods). Co-occurrence analysis identifies interconnectivity between contigs based on shared trends of abundances within samples. Group I and II contigs were associated with three separate co-occurrence networks, defined as distinct modules, in baseline MF viral communities from both VLP and IND samples (Figures 5a and b). The three modules were observed to contain similar contigs and co-occurring linkages, with the exception of the absence of a single contig (11070_5415_VLP) in the VLP co-occurrence network that was observed in the IND network. Module I was comprised of a total of 12 Group I and Group II contigs. These contigs were similar to phages known to be associated with Firmicutes (*Clostridia* and *Bacillus*) and Bacteroidetes as well as Caudovirales. The abundances of these contigs in both viral fractions were correlated to 16S rRNA genes- or BAC-associated sequences that shared similarity to sequences associated with Bacillales, Lactobacillales and Clostridiales. Module II contained contigs from Group I and II and was similar between VLP and IND fractions (18 contigs in VLP samples, 19 contigs in IND). Module II contigs were similar to phages previously known to be associated with Firmicutes (Bacilli) as well as Caudiovirales (Siphoviridae). In both fractions, these contigs were significantly correlated to 16S rRNA amplicons or BAC contigs that were similar to sequences associated with Erysipelotrichales and Clostridiales.

## Discussion

### Unprecedented access to the gut microbiome through multiple targeted sequencing efforts

Recent insights into the microbial processes and pathways that contribute to gut microbiome stability and resilience have focused on bacterial responses to diet in the gut system (Zhang *et al.*, 2012; David *et al.*, 2013). Despite their abundance in the gut microbiome (Breitbart *et al.*, 2008; Reyes *et al.*, 2010; Minot *et al.*, 2011), there have been few efforts to characterize the role of viruses alongside their bacterial partners in the dynamics of the gut microbiome.

In this study, metagenomic sequencing approaches were not only used to evaluate bacterial hosts and their response to dietary perturbations but also the viruses alongside these hosts. To investigate the lifestyles of phages in gut microbiome faecal samples, metagenomes were experimentally partitioned to access communities of both extracellular virus particles and induced prophages. Overall, we observed diverse viral communities living alongside gut bacterial communities, and consistent with previous studies, found that both bacterial and viral communities are diet-specific. The high diversity of both bacterial and viral communities in our samples supports complex phage-bacterial interactions within the gut microbiome, which is consistent with previous observations in artificial communities in gnotobiotic mice (Reyes *et al.*, 2013).

As expected, the BAC samples were mostly comprised of sequences related to bacterial genes with a broad diversity of functions, whereas the viral metagenomes were largely dominated by sequences related to phage metabolism. Owing to both our experimental design to target viral fractions as well as the smaller size of viral genomes relative to bacterial genomes, our cumulative reference assembly comprised a large fraction of reads originating from the viral fractions (for example, 82–92% of VLP and IND reads aligned to the final assembly) and a smaller proportion of reads from the bacterial fraction (7–9%). To complement the relatively shallow sequencing depth of metagenomes from the bacterial fraction (for example, <10% of reads mapped), 16S rRNA amplicon sequencing was performed to provide additional information about the structure of the bacterial gut community throughout the experiment. Notably, in our assembled reference, the large majority of sequences could not be classified against known sequences. Many of these unknown sequences originated from viral fractions, highlighting the need to develop improved references for characterizing these communities. These sequences reveal a significant community in these microbiomes that are uncharacterized and that are overlooked by relying on previously characterized reference genomes. Overall, among viral contigs, we observed that viruses were largely temperate, evidenced in the overlapping presence of similar contigs in both IND and VLP fractions (Figure 5) and consistent with previous reports (Reyes *et al.*, 2010; 2013). Contigs identified in both fractions were associated with phage integration and excision, packaging machinery, capsid proteins, entry and exit and phage regulation of gene expression.

The combination of the three fractions served as an effective strategy for assessing changes in community diversity at coarse time resolution (for example, every few weeks). Together with weekly 16S rRNA bacterial community data, we could explore responses of the bacterial and viral community structures, as well as functional responses, to diet changes, specifically, their resistance to diet disturbances. As the low viral biomass available in mouse stool required the use of whole-genome amplification, we expect biases in quantification of sequence abundance. Nevertheless, our biological replicates exhibited similar responses, suggesting that any amplification biases are minimal or are similar across samples. Despite these challenges, the resulting sequencing catalog generated from this effort is an important reference for future studies.

### Responses of microbiome communities to diet

We observed significantly different bacterial communities in response to diet shifts, and this result is consistent with previous reports that have indicated diet-specific changes in gut bacterial communities (Turnbaugh, Ridaura, *et al.*, 2009; Zhang *et al.*, 2012). Similar to previous studies (Carmody *et al.*, 2015), we observed increases in the numbers of Firmicutes, and in particular, increases in members of the order Clostridiales, in mice fed the high MF, high-sugar (MF) diet. Interestingly, based on 16S rRNA amplicons, the relative abundance of Turicibacterales increased significantly in baseline MF mice during the diet switch (*P* =0.0039). In baseline LF mice, the mean relative abundance of this taxon decreased, though not significantly, when on the MF diet (mean relative abundance, Day 0 vs Day 22, 5.5% vs 1.9%). These diet-specific changes associated with Turicibacterales have also been reported by others, including reduced relative abundances of *Turicibacter* in mice fed a high-fat diet (Everard *et al.*, 2014) and mice fed a high-resistant starch diet (Tachon *et al.*, 2013), and may indicate their sensitivity to dietary components. We also observed rapid changes in both bacterial and viral gut communities occurred within 24 h after dietary shifts, which is consistent with previous work showing rapid changes in the microbiomes of mice following dietary perturbation in bacteria (Turnbaugh, Ridaura, *et al.*, 2009; McNulty *et al.*, 2013) and viruses (Minot *et al.*, 2011), and illustrates the sensitivity of the gut microbiome to dietary change. In the baseline LF mice, we observed an increase in alpha diversity of the bacterial communities on Day 22, in contrast to the decrease in bacterial diversity and richness observed by Zhang *et al.* (2012) in their study's high-fat fed treatment group. The diets in our study contained comparable amounts of carbohydrates (LF, 13.8% and MF, 15.8%), whereas in Zhang *et al.*'s study, the amount of carbohydrates in the HFD diets was much lower than in the NC diet (26.3% compared with 61.3%). These dietary differences may have contributed to the contrasting changes in bacterial diversity depending on how certain bacterial members responded to these specific dietary components. In BAC metagenomes, we observed significantly different bacterial communities but not different functional profiles on Days 0, 22 and 43 of the experiment. The resistance of these gut functions to diet changes likely contributes to overall stability, where various gut bacteria likely share redundant functions.

In evaluating the resistance of various communities to the diet shift, we compared communities in two contrasting diet treatments at various time points. To assess the resistance of these communities to its corresponding diet shift, we compared mainly baseline (Day 0) and washout communities (Day 43). In contrast to bacterial communities that were similar before and after the diet shift, virus communities were significantly different, most profoundly observed in the baseline MF IND and VLP metagenomes. This result, supported by direct counts of VLPs, contrasts with previous reports of similar bacterial and viral trends in response to a diet shift (Minot *et al.*, 2011). These data suggest that diet history has a role in virome responses and that some members of the virome may fail to recover from a perturbation, just as some bacterial members of the gut microbiota demonstrate hysteresis after dietary oscillations (Carmody *et al.*, 2015).

To identify potential drivers in the baseline MF virus fractions that were associated with the observed unique responses, we identified contigs that were observed to change significantly in relative abundance over the experiment in VLP and IND fractions. The most significant functions were associated with known phage-associated sequences, and these contigs were grouped into two representative clusters based on their abundances in baseline MF viral fractions. The majority of these contigs were similar to sequences originating from phages associated with Firmicutes, both Clostridia and Bacilli. Firmicutes have previously been identified as a dominant division responding to diet in the gut microbiome (Turnbaugh *et al.*, 2006; Turnbaugh, Hamady, *et al.*, 2009), and the identification of viruses associated with this phylum suggests that these communities have an important role in virus–bacteria dynamics. This finding is also consistent with previous observations of the abundant Firmicutes-associated phages in gut microbiome studies (Minot *et al.*, 2011). Phages associated with the tailed bacteriophage order, Caudovirales, were also identified as significantly changing in abundance within baseline MF viral fractions. These primarily temperate phages have recently also been observed to be enriched in Crohn's disease and ulcerative colitis patients (Norman *et al.*, 2015) and speculated to have a role in inflammatory bowel diseases. In co-occurrence network analyses, Caudovirales-associated contigs were observed to correlate with Bacilli-associated viruses and bacteria, including Erysipelotrichales and Clostridiales, two of the taxa that significantly changed in abundance in our baseline LF mice. Both of these potential bacterial hosts have been observed to decrease in abundance in correlation with inflammatory bowel diseases (Gevers *et al.*, 2014; Rooks *et al.*, 2014). Overall, these results indicate the presence of multiple and diverse potential bacteria correlated with viruses that drive the response of the baseline MF communities to a diet shift and indicates that virus–bacteria dynamics of the gut microbiome are complex: phages could potentially be associated with multiple hosts or specific virus–bacteria dynamics could indirectly impact diverse bacterial communities. Interestingly, in both the baseline MF VLP and IND fractions, gene abundance network co-occurrence patterns were similar, providing further evidence of the temperate lifestyle of gut microbiome viruses.

To our knowledge, this is the first demonstration that viral communities can respond to specific diet perturbations independently and that bacteria and viruses in the same mice may have unique resistance to disturbances. The contrasting responses of the baseline MF viral communities relative to their counterpart LF viral communities suggest that historical contingency—not just the current diet treatment of the host—may have a key role in community development; for example, some factor during the early life of the baseline MF mice may have constrained their microbiomes' responses to the LF diet and to the reversion to MF. Importantly, our experimental design cannot rule out the environmental differences resulting from housing the baseline LF and MF mice in different cages, often a problem due to the logistics of housing in mammalian studies (Campbell *et al.*, 2012). Nonetheless, distinct responses were observed in bacterial and viral communities in contrasting environments, where a substantial difference was diet treatment. Interestingly, we found decreases in the VLP counts from baseline LF mice at time points when alpha diversity of the bacterial communities increased. The differences in the responses resemble the contrasting responses of the bacterial and viral communities observed in Crohn's disease patients, in which increased diversity of bacteriophages were found alongside bacterial communities with decreased diversity (Norman *et al.*, 2015). This study provides consistent evidence that viral and bacterial communities can develop independently and provide important rationale for further investigation of viruses in these systems. In addition, this study also suggests that dietary history can have a distinct impact on the functional profile of the virome, and these viral dynamics may contribute to community shifts, including potentially dysbiotic shifts, in the gut microbiome. Though this study demonstrates the importance for understanding viral dynamics in the gut, like many metagenomic studies, it leads to more questions than answers: would baseline MF viruses trend toward recovery over a longer time period? How quickly can viral communities respond and recover? What are the most critical host–phage relationships for community stability? What are the mechanisms driving the contrasting baseline MF virus response? To further expand toward our understanding of long-term stability and dynamics of viral communities in the gut, improved methods combined with high resolution longitudinal studies are much needed to understand rates of virus-host infection, in tractable mouse model systems, such as in gnotobiotic mice (Reyes *et al.*, 2013).

## Figures and Tables

**Table 1:** Composition of low- and high-milkfat experimental diets

	*Low fat (AIN 93)*	*Milk fat (TD.97222)*
kcal g^−1^	3.6	4.4
% carbohydrate	13.8	15.8
% protein	76	46.8
% fat	10.2	37.4
Casein	0.14	0.195
l-cysteine	0.0018	—
DL-methionine	—	0.003
Corn starch	0.461	0.13
Maltodextrin	0.155	0.14
Sucrose	0.1	0.255
Soybean oil	0.04	—
Anhydrous milkfat	—	0.18
Cellulose	0.05	0.045
Mineral mix	0.035	0.035
Vitamin mix	0.0145	0.01
Choline bitartrate	0.0028	0.001
Antioxidant	0.000008	0.00004

**Table 2:** Sequencing yield (Mbp) of BAC, IND and VLP metagenomes

	*BAC*	*IND*	*VLP*	*Cumulative*
Baseline LF	50 276	27 387	34 050	111 713
Baseline MF	63 321	19 146	16 400	98 867

Abbreviations: BAC, bacterial; IND, induced prophage; LF, low-fat diet; MF, milkfat diet; VLP, virus-like particles.

**Table 3:** Assembled contigs from the BAC, IND, VLP and merged microbiome metagenomes

	*Total contigs*	*Total length (bp)*	*Largest contig (bp)*
BAC contigs	8736	2 372 344	5505
IND contigs	104 036	73 108 696	121 514
VLP contigs	16 273	33 051 399	86 772
Final contigs	117 460	100 224 821	121 514

Abbreviations: BAC, bacterial; IND, induced prophage; VLP, virus-like particles.

**Table 4:** Analysis of Bray–Curtis dissimilarity among bacterial and viral communities

	*Baseline LF*				*Baseline MF*			
	*BAC*	*VLP*	*IND*	*16S*	*BAC*	*VLP*	*IND*	*16S*
Dav 0 vs 1	—	0.3	0.001389	0.1034	—	0.1	0.2	1
Day 0 vs 22	0.001389*	0.001389*	0.1	0.094	0.2	0.001389*	0.001389*	0.088
Day 0 vs 43	0.1	0.5014	0.1	0.089	0.6	0.001389*	0.001389*	0.428

Abbreviations: BAC, bacterial; IND, induced prophage; LF, low-fat diet; MF, milkfat diet; VLP, virus-like particles. Asterisks indicate *P*-values of pairwise ADONIS comparisons that are significant after Bonferroni correction.
