# Correction to: Divergent responses of viral and bacterial communities in the gut microbiome to dietary disturbances in mice

**DOI:** 10.1093/ismejo/wrag024

**Published:** 2026-02-19

This is a correction to: Adina Howe, Daina L Ringus, Ryan J Williams, Zi-Ning Choo, Stephanie M Greenwald, Sarah M Owens, Maureen L Coleman, Folker Meyer, Eugene B Chang, Divergent responses of viral and bacterial communities in the gut microbiome to dietary disturbances in mice, *The ISME Journal*, Volume 10, Issue 5, May 2016, Pages 1217–1227, https://doi.org/10.1038/ismej.2015.183

In the original publication there were errors in Table 1 and within the text. These error are the values for the composition of the % carbohydrates and % protein.

The correct amounts are: for the % of carbohydrate “46.8”; and for the % of protein “15.8”.

These errors are outlined only in this correction notice to preserve the version of record.

