# Supplementary material for: Divergent responses of viral and bacterial communities in the gut microbiome to dietary disturbances in mice
Source: ISME J. 2015 Oct 16;10(5):1217–27. doi: 10.1038/ismej.2015.183 (PMC5029215; doi:10.1038/ismej.2015.183)
Supplement: Supplementary Figures [file ismej2015183x1.docx]

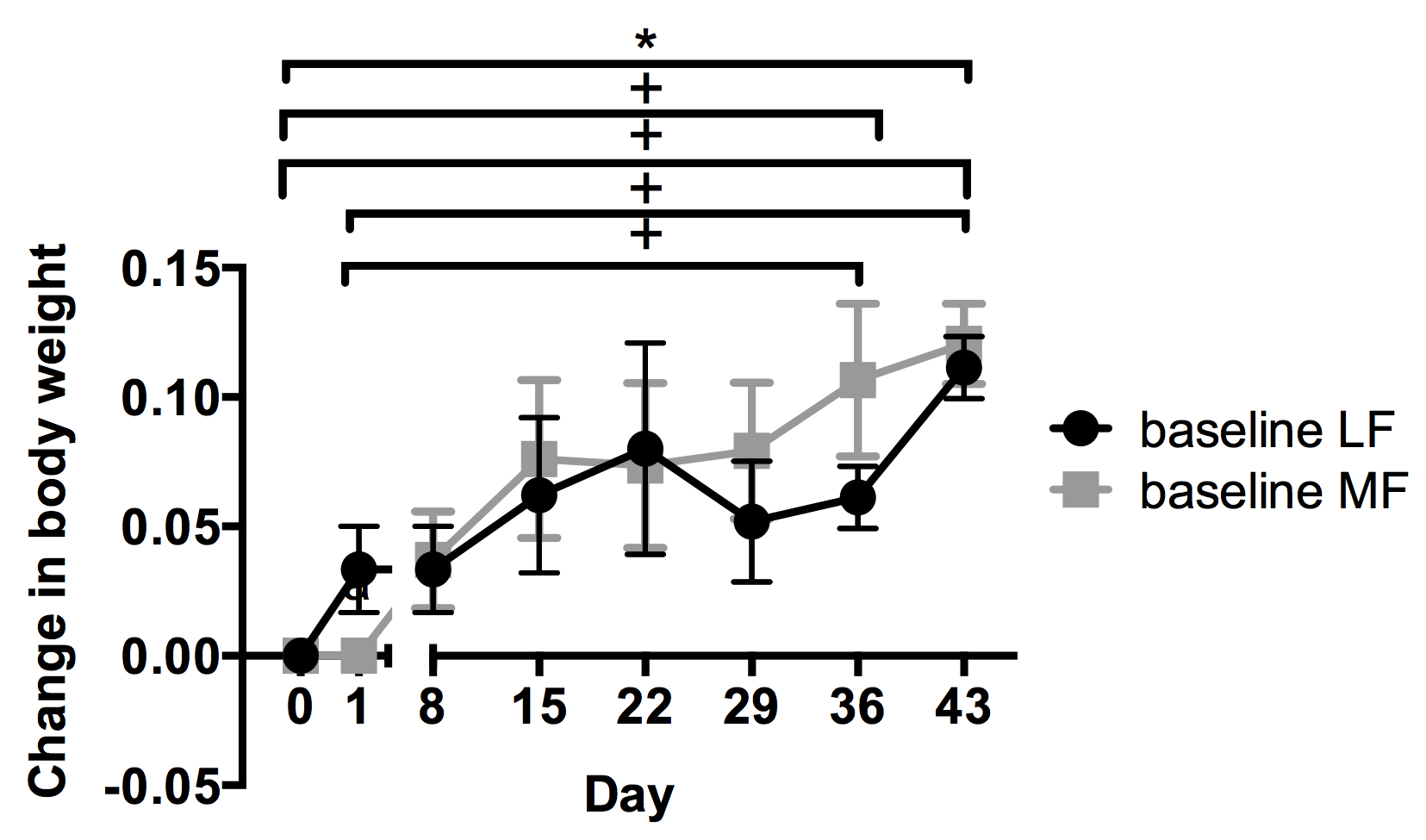


Figure S1. Changes in body weights of mice over six-week study calculated as increase or decrease in body weight as a percentage of initial body weight at Day 0. Data are shown as mean ± S.E.M. Two-way ANOVA was performed on percent changes in weight between groups on each day; for all comparisons p > 0.05. Two-way ANOVA was performed within groups comparing percent changes on different days. For baseline LF mice, * = P < 0.05; for baseline MF mice, + = P < 0.05.


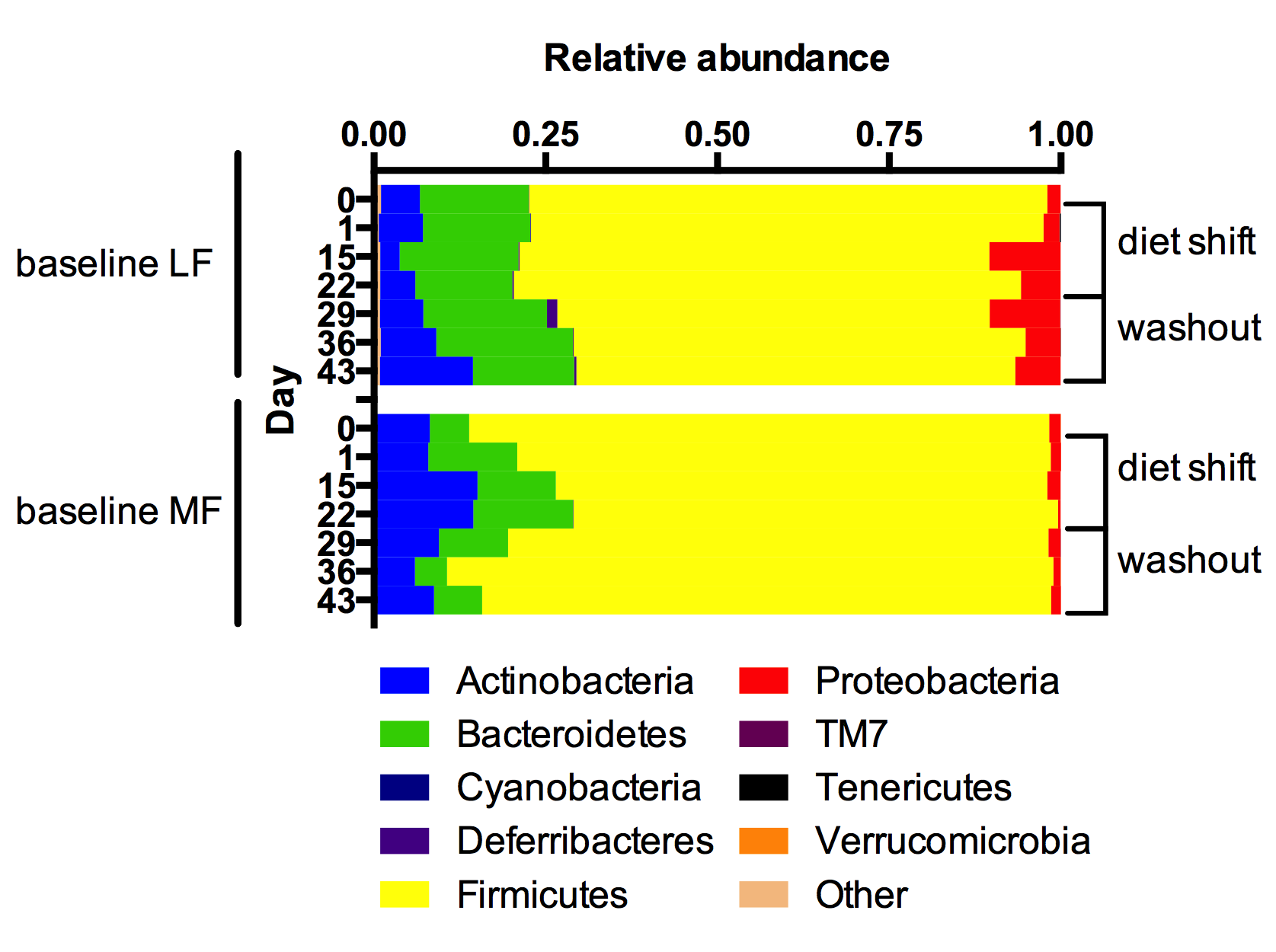


Figure S2. Relative abundance plots of phylum-level taxonomic classification from 16S rRNA gene amplicon analysis. Standardized mean relative abundances of phyla distribution from samples within each treatment group from the indicated day are shown.


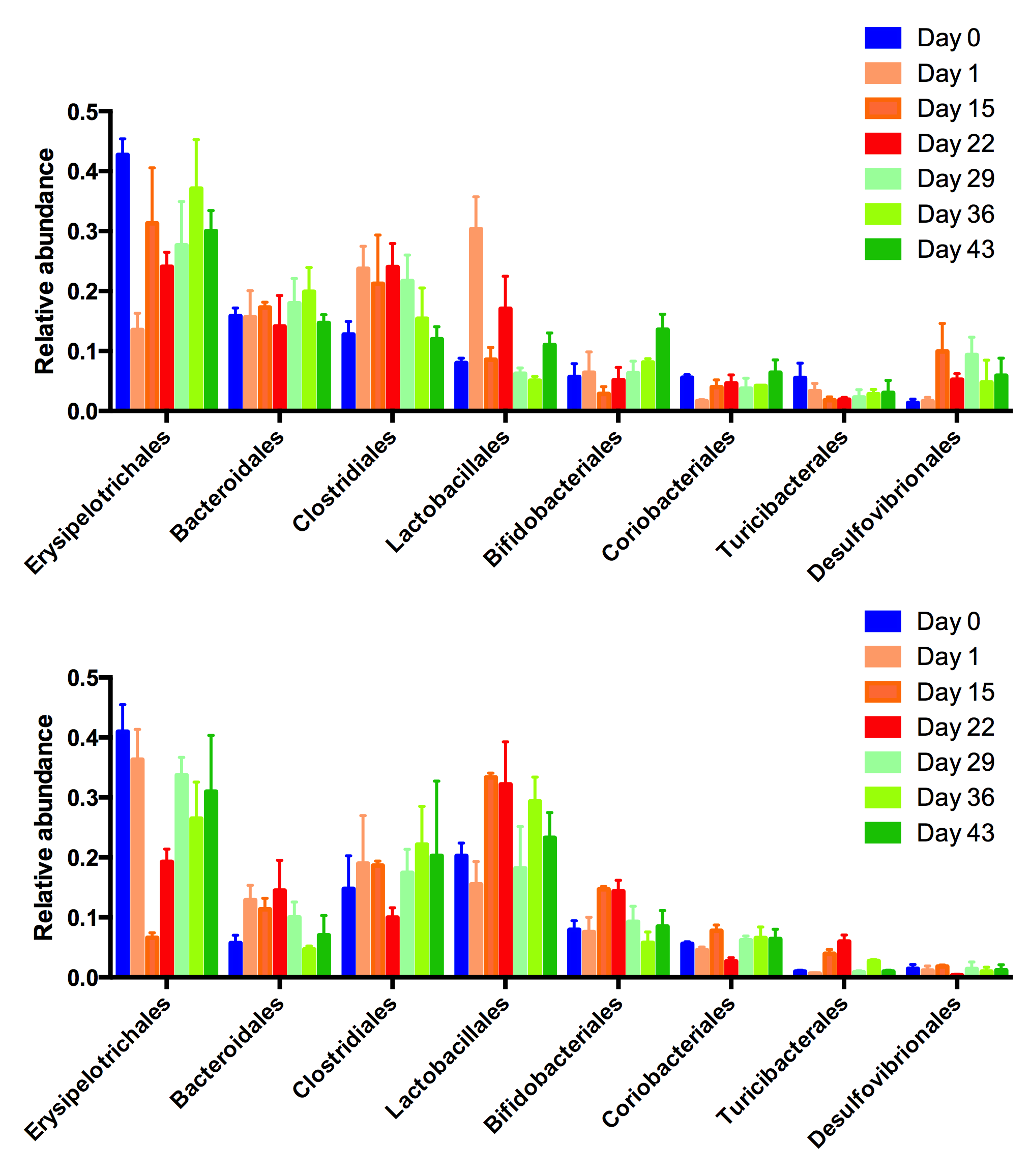


**A**

**B**

Figure S3. Analysis of bacterial community structure through 16S rRNA amplicon analysis. Mean relative abundances of order level taxa in fecal bacterial communities present at > 1% in A) baseline LF and B) baseline MF mice. Data are shown as mean ± S.E.M.


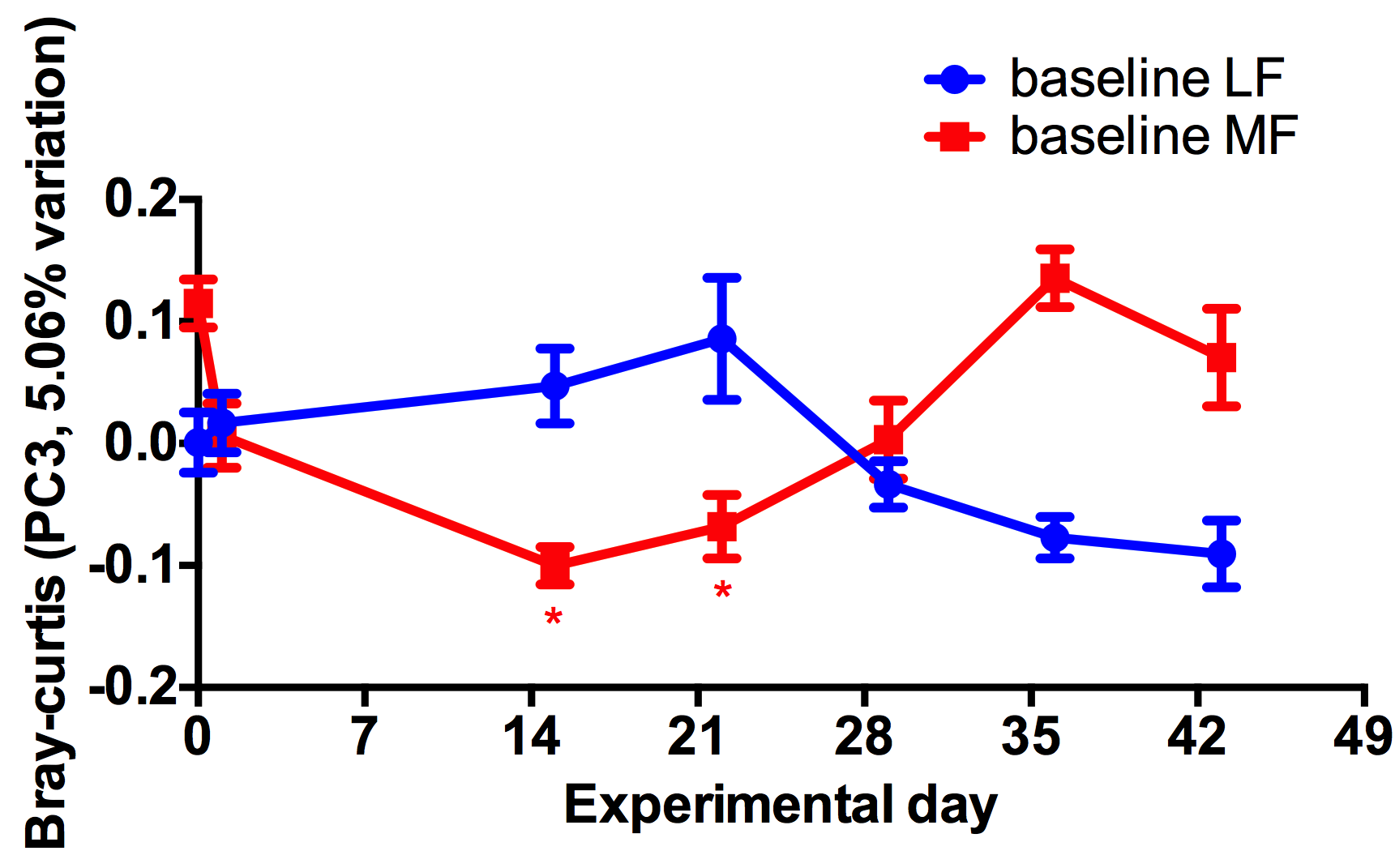


Figure S4. Distances of bacterial communities from baseline communities (based on the third principle coordinate axis of Bray-Curtis PCOA plot in Fig. 3F). Mean pairwise distances ± S.E.M of baseline LF and baseline MF communities of individual mice between each time point and Day 0. The asterisk represents significant differences at each time point relative to Day 0 (P-value: * < 0.05, Two-way ANOVA, Bonferroni correction for multiple comparisons).

Figure S5. NMDS of Bray-Curtis distances of sequence abundances in BAC, IND, and VLP metagenomes (NMDS Stress 0.1380967). Sample colors represent fraction of origin, and sample shapes represent different treatment groups.


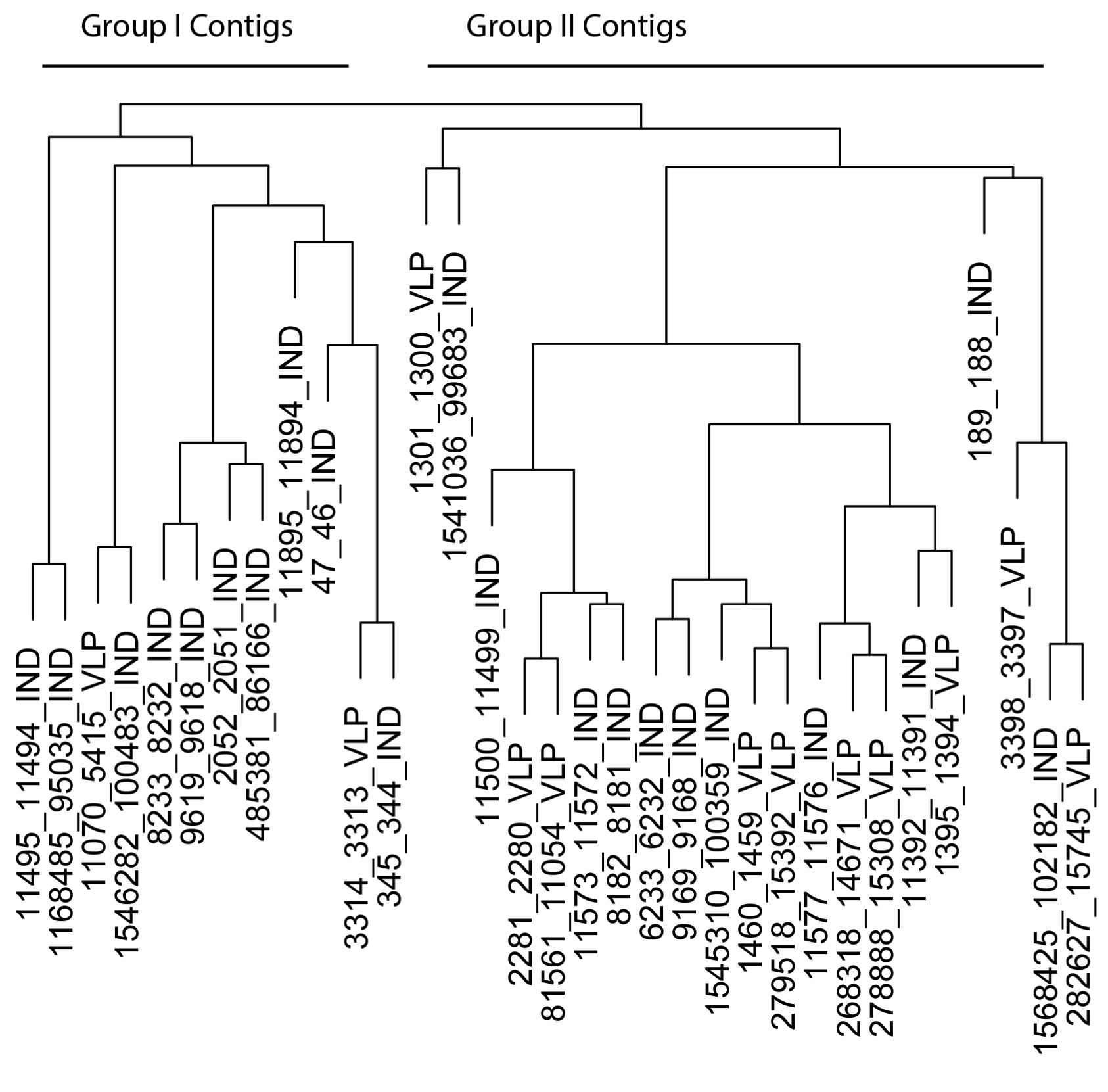


Figure S6. Similarity hierarchical clustering (Bray-Curtis distances of relative abundances) of contigs that changed significantly over the experiment. These contigs shared sequence similarity to known proteins associated with phage, prophages, transposable elements, and plasmids.

Figure S7. Estimated abundances of contigs that were present in baseline MF IND samples (averaged for all replicate samples).  Group I and Group II clustered contigs are shown in red and blue, respectively.

Figure S8. Estimated abundances of contigs that were present in baseline MF VLP samples (averaged for all replicate samples).  Group I and Group II clustered contigs are shown in red and blue, respectively.

Figure S9. Estimated abundances of contigs that were present in baseline LF IND samples (averaged for all replicate samples).   Group I and Group II clustered contigs are shown in red and blue, respectively.

Figure S10. Estimated abundances of contigs that were present in baseline LF VLP samples (averaged for all replicate samples).   Group I and Group II clustered contigs are shown in red and blue, respectively.
