# Supplementary material for: Divergent responses of viral and bacterial communities in the gut microbiome to dietary disturbances in mice
Source: ISME J. 2015 Oct 16;10(5):1217–27. doi: 10.1038/ismej.2015.183 (PMC5029215; doi:10.1038/ismej.2015.183)
Supplement: Supplementary Text [file ismej2015183x2.docx]

Supplementary Methods

**Virus-like particles (VLP) and prophage (IND) isolation, quantification, and DNA extraction**

Stool was weighed and homogenized in 0.5 ml SM buffer (100 mM NaCl, 8 mM MgSO_4_, 50 mM Tris-HCl, pH 7.5) by vortexing. Stool slurries were centrifuged (2,700 *x g*, 20 min, 4^o^C) to pellet debris. To enrich for virus-like particles (VLPs), the resulting supernatants were filtered through a 0.22 μm syringe filter (Millipore), pooled by mouse, and adjusted to 10% iodixanol (Optiprep; Axis-Shield) to a final volume of 7.4 ml. The filtrate was layered onto a 4 ml step iodixanol gradient (20, 30, 40, 50% v/v in SM buffer) and centrifuged at 25,000 rpm for 2 hours at 4^o^C in a SW41 swinging bucket rotor (Beckman). An aliquot (1.1 ml) was collected by inserting a syringe below the 40% layer. For prophage induction, the remaining stool slurry pellet was resuspended in 1 ml pre-reduced thioglycollate broth (USP alternative; Sigma Aldrich) with 1 μg/ml mitomycin C and incubated anaerobically at 37°C for 20 hr. We chose mitomycin C for induction because it is a common inducer of phages in gut-related taxa of bacteria and because the opacity of the fecal slurries might prevent equal exposure of bacterial communities to an inducer by using other methods, i.e., UV induction (Abratt *et al.*, 1985; Klieve *et al.*, 1989). Induced prophages (IND) were isolated from VLPs collected from mitomycin C-incubated slurries as described for VLPs above. For VLP and IND fractions, after collection of VLPs from the density gradient, chloroform (200 μl) was added, the solutions were centrifuged (17,000 *x g*, 5 min, room temp.), and the virus-containing supernatants retained. To estimate abundance of virus particles, aliquots (50 μl) of density gradient purified VLP fractions were stained with SYBR Gold and imaged by confocal fluorescence microscopy at the University of Chicago Integrated Light Microscopy Facility to calculate VLPs/g of mouse stool (wet weight) (Thurber *et al.*, 2009). VLPs were concentrated using 100 kDa MWCO centrifugation filters (Amicon) and washed twice with SM buffer to remove iodixanol. Concentrated VLPs were treated with DNase I (Sigma Aldrich, 80 Units ml^-1^) for 3 hr at 37^o^C and the enzyme was heat inactivated at 65°C for 15 min. PCR to amplify the bacterial 16S gene using universal primers (27F and 1525R; (Lane, 1991))was performed on DNA isolated from DNase-treated VLPs to confirm the absence of bacterial contamination.

Virus DNA was extracted from DNase I-treated samples using the QIamp MinElute Virus Spin kit, following manufacturer’s instructions but without the use of carrier RNA. DNA from viral fractions (VLP and IND) was amplified by whole genome amplification (GenomiPhiV2; GE Lifesciences) for 18 hr in duplicate per sample, then pooled and purified using the DNeasy Blood & Tissue kit. Total DNA for BAC fractions was extracted from the other remaining stool pellet as described (Huang *et al.*, 2013) and purified using the Qiagen DNeasy Blood & Tissue kit.

**16S rRNA-based amplicon library preparation and data analysis**

The V4-V5 region of the 16S rRNA encoding gene was amplified using standard Earth Microbiome Project protocols (http://www.earthmicrobiome.org/emp-standard-protocols/, 515F and 806R primers). Sequencing was performed at the High-Throughput Genome Analysis Core at Argonne National Laboratory. Single end sequences (150 bp) were then trimmed and classified using the Quantitative Insights into Microbial Ecology (QIIME) toolkit (Caporaso, Kuczynski, *et al.*, 2010) OTUs were clustered at 97% sequence identity using open reference OTU picking protocol (QIIME) against the Greengenes database (05/13 release) (McDonald *et al.*, 2012). These representative sequences were aligned using PyNAST (Caporaso, Bittinger, *et al.*, 2010) and taxonomy was assigned to them using the RDP Classifier (Wang *et al.*, 2007). The PyNAST-aligned sequences were also used to build a phylogenetic tree with FastTree (Price *et al.*, 2010) and Bray-Curtis distances were computed in order to produce a beta-diversity dissimilarity matrix. For alpha and beta diversity analyses, OTU tables were normalized by a cumulative sum scaling approach using the metagenomeSeq package (Paulson *et al.*, 2013).

**Assembly, abundance, and annotation of metagenomes**

Paired-end sequencing libraries were prepared from DNA originating from the BAC, VLP, and IND fractions. Sequencing libraries were prepared by Argonne National Laboratory with the PrepX NGS Library Kit (IntegenX Inc.) with an average insert size of 180 bp and sequenced on an Illumina HiSeq2000 platform. For assembly, sequencing reads for each fraction were pooled, resulting in a total of three assemblies. Illumina adapters were removed from resulting sequences using Trimmomatic with the parameter ILLUMINACLIP:2:30:10 (Bolger *et al.*, 2014). Paired-end sequences were also merged with PandaSeq (Masella *et al.*, 2012)with default settings. The resulting single and paired sequences were then normalized as previously described (Howe *et al.*, 2014) with the following settings: (K=20, C=20, x=50e9, N=4). Low abundance k-mers were removed from the *khmer* package (v1.0), using counting bloom filters from the previous normalization step and removing k-mers below a coverage of 2 with the variable coverage option. Resulting normalized reads were further partitioned as described previously (Howe *et al.*, 2014) using the following settings: K=120, x=10e9, N=4, subset size = 1e5. Extracted partitions were assembled as previously described (Howe *et al.*, 2014) using Velvet assembler (1.2.10, (Zerbino & Birney, 2008)) using odd k-mer lengths ranging from 33 to 69. Resulting assembled contigs were merged as described previously in (Howe *et al.*, 2014) using CD-HIT (v4.6,(Fu *et al.*, 2012)) and Minimus2 (Amos v3.1.0, (Sommer *et al.*, 2007)). Coverage of all contigs was estimated with the median bp alignments of untrimmed reads using bowtie2 (v4.7.2 (Langmead & Salzberg, 2012)) (--sensitive).

The accuracy of assemblies and estimated representation of assembled sequences in each metagenome were evaluated by aligning all sequencing reads from each sample to assembled contigs. Contigs assembled from the bacterial and viral fractions (combination of VLP and IND) were associated with 0.2% and 7.8%, respectively, of read alignments being mapped discordantly. Overall, high sequencing coverage (as evaluated by proportion of reads mapped) was achieved for VLP and IND metagenomes, with 82-92% of reads originating from viral fractions mapped to assembled contigs (Table S2). For comparisons, the estimated coverage in each sample was standardized by total coverage. Assembled sequences greater than 200 bp were annotated using the Metagenomics RAST (MG-RAST, v3.3.7.3, (Meyer *et al.*, 2008)) server. Functional annotations were obtained from the Subsystems source database with the following criteria: identity >= 60%, length >= 15 aa, e-value <= 1e-5. Taxonomy was obtained from the taxonomic ID associated with RefSeq identifiers linked to MG-RAST best match sequence. If more than one taxa was associated with a sequence, one was randomly selected to be representational for the sequence throughout the analysis. Contigs were screened for contamination of sequences, resulting in 0.19% of VLP contigs and 2.4% of IND contigs sharing similarity to the genus *Mus* (M5NR database), and these contigs were not included in our analysis.

**Co-occurrence network analysis of specific contigs**

Co-occurrence networks to determine whether or not certain VLP contigs had positive relationships with 16S rRNA and BAC metagenomes were performed as previously described (Williams *et al.*, 2014). Spearman’s correlations were generated for all pairs of sequence data (specific contigs identified as significant, Table S7) using the rcorr() function from the Hmisc package in R (Harrell, 2015). P-values were adjusted using a false discovery rate (Pike, 2011)through the fdrtool() function in the fdrtool package in R (Klaus & Strimmer, 2013), where Q-values < 0.05 were considered in the network. This value was considered as an appropriate cut-off as all correlations had a *rho* value greater than 0.83; therefore, we did not consider multiple cut-offs as suggested in (Williams *et al.*, 2014). The resulting network was constrained to only include nodes that were directly adjacent to specific contigs. Networks were generated using the graph.edgelist() function from the igraph package in R (Csardi & Nepusz, 2006), and a combination of the intergraph and GGally packages were used to visualize the network (Schloerke *et al.*, 2011) (Bojanowski, 2015).

**PCR confirmation of contigs**

PCR was performed to confirm the presence of contigs in the BAC, VLP, and IND DNA samples. Primers used are listed in Table S8.

**References**

Abratt VR, Jones DT, Woods DR. (1985). Isolation and physiological characterization of mitomycin C-sensitive/UV-sensitive mutants in *Bacteroides fragilis*. *J Gen Microbiol* **131**:2479–2483.

Bojanowski M. (2015). Coercion Routines for Network Data Objects. R package version 2.0-2. *mbojangithubio*. http://mbojan.github.io/intergraph (Accessed July 21, 2015).

Bolger AM, Lohse M, Usadel B. (2014). Trimmomatic: a flexible trimmer for Illumina sequence data. *Bioinformatics* **30**:2114–2120.

Caporaso JG, Bittinger K, Bushman FD, DeSantis TZ, Andersen GL, Knight R. (2010). PyNAST: a flexible tool for aligning sequences to a template alignment. *Bioinformatics* **26**:266–267.

Caporaso JG, Kuczynski J, Stombaugh J, Bittinger K, Bushman FD, Costello EK, *et al.* (2010). QIIME allows analysis of high-throughput community sequencing data. *Nat Methods* **7**:335–336.

Csardi G, Nepusz T. (2006). The igraph software package for complex network research. *InterJournal, Complex Systems* **1695**:1–9.

Fu L, Niu B, Zhu Z, Wu S, Li W. (2012). CD-HIT: accelerated for clustering the next-generation sequencing data. *Bioinformatics* **28**:3150–3152.

Harrell FE Jr. (2015). Hmisc: Harrell Miscellaneous. R package version 3.16-0.

Howe AC, Jansson JK, Malfatti SA, Tringe SG, Tiedje JM, Brown CT. (2014). Tackling soil diversity with the assembly of large, complex metagenomes. *Proc Natl Acad Sci U S A* **111**:4904–4909.

Huang EY, Leone VA, Devkota S, Wang Y, Brady MJ, Chang EB. (2013). Composition of dietary fat source shapes gut microbiota architecture and alters host inflammatory mediators in mouse adipose tissue. *JPEN J Parenter Enteral Nutr* **37**:746–754.

Klaus B, Strimmer K. (2013). fdrtool: Estimation of (Local) False Discovery Rates and Higher Criticism. R package version 1.2.13.

Klieve AV, Hudman JF, Bauchop T. (1989). Inducible bacteriophages from ruminal bacteria. *Appl Environ Microbiol* **55**:1630–1634.

Lane DJ. (1991). 16S/23S rRNA sequencing. In:*Nucleic acid techniques in bacterial systematics*, Stackebrandt, E & Goodfellow, M (eds), John Wiley & Sons: Chichester, England, pp. 115–175.

Langmead B, Salzberg SL. (2012). Fast gapped-read alignment with Bowtie 2. *Nat Methods* **9**:357–359.

Masella AP, Bartram AK, Truszkowski JM, Brown DG, Neufeld JD. (2012). PANDAseq: paired-end assembler for illumina sequences. *BMC Bioinformatics* **13**:31.

McDonald D, Price MN, Goodrich J, Nawrocki EP, DeSantis TZ, Probst A, *et al.* (2012). An improved Greengenes taxonomy with explicit ranks for ecological and evolutionary analyses of bacteria and archaea. *ISME J* **6**:610–618.

Meyer F, Paarmann D, D'Souza M, Olson R, Glass EM, Kubal M, *et al.* (2008). The metagenomics RAST server–a public resource for the automatic phylogenetic and functional analysis of metagenomes. *BMC Bioinformatics* **9**:386.

Paulson JN, Stine OC, Bravo HC, Pop M. (2013). Differential abundance analysis for microbial marker-gene surveys. *Nat Methods* **10**:1200–1202.

Pike N. (2011). Using false discovery rates for multiple comparisons in ecology and evolution. *Methods Ecol Evol* **2**:278–282.

Price MN, Dehal PS, Arkin AP. (2010). FastTree 2–approximately maximum-likelihood trees for large alignments. *PLoS One* **5**:e9490.

Schloerke B, Crowley J, Cook D, Hofmann H, Wickham H, Briatte F, *et al.* (2011). Ggally: Extension to ggplot2. R package version 0.5.0.

Sommer DD, Delcher AL, Salzberg SL, Pop M. (2007). Minimus: a fast, lightweight genome assembler. *BMC Bioinformatics* **8**:64.

Thurber RV, Haynes M, Breitbart M, Wegley L, Rohwer F. (2009). Laboratory procedures to generate viral metagenomes. *Nat Protoc* **4**:470–483.

Wang Q, Garrity GM, Tiedje JM, Cole JR. (2007). Naive Bayesian classifier for rapid assignment of rRNA sequences into the new bacterial taxonomy. *Appl Environ Microbiol* **73**:5261–5267.

Williams RJ, Howe A, Hofmockel KS. (2014). Demonstrating microbial co-occurrence pattern analyses within and between ecosystems. *Front Microbiol* **5**:1–10.

Zerbino DR, Birney E. (2008). Velvet: algorithms for de novo short read assembly using de Bruijn graphs. *Genome Res* **18**:821–829.
